# Ethnic and socioeconomic disparities in initiation of second‐line antidiabetic treatment for people with type 2 diabetes in England: A cross‐sectional study

**DOI:** 10.1111/dom.14874

**Published:** 2022-11-02

**Authors:** Patrick Bidulka, Rohini Mathur, David G. Lugo‐Palacios, Stephen O'Neill, Anirban Basu, Richard J. Silverwood, Paul Charlton, Andrew Briggs, Liam Smeeth, Amanda I. Adler, Ian J. Douglas, Kamlesh Khunti, Richard Grieve

**Affiliations:** ^1^ Department of Non‐Communicable Disease Epidemiology London School of Hygiene & Tropical Medicine London UK; ^2^ Department of Health Services Research and Policy London School of Hygiene & Tropical Medicine London UK; ^3^ The Comparative Health Outcomes, Policy & Economics (CHOICE) Institute University of Washington School of Pharmacy Seattle Washington; ^4^ Centre for Longitudinal Studies, UCL Social Research Institute University College London London UK; ^5^ Patient Research Champion Team National Institute for Health Research Twickenham UK; ^6^ Diabetes Trials Unit, The Oxford Centre for Diabetes, Endocrinology and Metabolism University of Oxford Headington UK; ^7^ Diabetes Research Centre University of Leicester Leicester UK

**Keywords:** ethnicity, oral antidiabetics, pharmacoepidemiology, socioeconomic deprivation, type 2 diabetes

## Abstract

**Aims:**

To assess any disparities in the initiation of second‐line antidiabetic treatments prescribed among people with type 2 diabetes mellitus (T2DM) in England according to ethnicity and social deprivation level.

**Materials and methods:**

This cross‐sectional study used linked primary (Clinical Practice Research Datalink) and secondary care data (Hospital Episode Statistics), and the Index of Multiple Deprivation (IMD). We included people aged 18 years or older with T2DM who intensified to second‐line oral antidiabetic medication between 2014 and 2020 to investigate disparities in second‐line antidiabetic treatment prescribing (one of sulphonylureas [SUs], dipeptidyl peptidase‐4 [DPP‐4] inhibitors, or sodium‐glucose cotransporter‐2 [SGLT2] inhibitors, in combination with metformin) by ethnicity (White, South Asian, Black, mixed/other) and deprivation level (IMD quintiles). We report prescriptions of the alternative treatments by ethnicity and deprivation level according to predicted percentages derived from multivariable, multinomial logistic regression.

**Results:**

Among 36 023 people, 85% were White, 10% South Asian, 4% Black and 1% mixed/other. After adjustment, the predicted percentages for SGLT2 inhibitor prescribing by ethnicity were 21% (95% confidence interval [CI] 19–23%), 20% (95% CI 18–22%), 19% (95% CI 16–22%) and 17% (95% CI 14–21%) among people with White, South Asian, Black, and mixed/other ethnicity, respectively. After adjustment, the predicted percentages for SGLT2 inhibitor prescribing by deprivation were 22% (95% CI 20–25%) and 19% (95% CI 17–21%) for the least deprived and the most deprived quintile, respectively. When stratifying by prevalent cardiovascular disease (CVD) status, we found lower predicted percentages of people with prevalent CVD prescribed SGLT2 inhibitors compared with people without prevalent CVD across all ethnicity groups and all levels of social deprivation.

**Conclusions:**

Among people with T2DM, there were no substantial differences by ethnicity or deprivation level in the percentage prescribed either SGLT2 inhibitors, DPP‐4 inhibitors or SUs as second‐line antidiabetic treatment.

## 1 INTRODUCTION

Most healthcare systems report inequities in disease incidence, healthcare delivery and outcomes according to people's socioeconomic status and ethnicity.^1^, ^2^ For countries with single‐payer systems such as England, national recommendations from agencies like the National Institute for Health and Care Excellence (NICE) encourage access to effective and cost‐effective interventions to maximize clinical benefit while also reducing health inequalities.^3^, ^4^, ^5^ Nonetheless in countries such as England, inequities in using healthcare interventions according to people's socioeconomic characteristics persist for diseases such as cardiovascular disease (CVD),^6^ chronic kidney disease (CKD),^7^ and type 2 diabetes mellitus (T2DM).^8^

There are inequities in T2DM prevalence and outcomes according to ethnicity^8^ and deprivation.^9^ People of Black and South Asian ethnicity, and people with lower income or lower educational attainment have a higher prevalence of T2DM, worse blood glucose control and earlier onset of macro‐ and microvascular complications compared with people of ethnicities other than Black and South Asian, higher incomes or higher educational attainment.^8^, ^9^, ^10^, ^11^, ^12^, ^13^ Ethnic minorities also tend to experience delays in T2DM treatment intensification when clinically indicated (therapeutic inertia),^12^ which may contribute to worse outcomes compared with White people.^14^, ^15^, ^16^, ^17^, ^18^ Other ethnic and socioeconomic inequities in T2DM treatment that could impact clinical outcomes, such as the type of second‐line antidiabetic treatment prescribed at treatment intensification from metformin monotherapy, are less well understood. Hence, we chose to examine the potential disparities in second‐line antidiabetic treatment prescribing by ethnicity and deprivation status.

For people with T2DM whose glycated haemoglobin (HbA1c) levels are poorly controlled, an important choice is which second‐line oral antidiabetic therapy to prescribe in addition to metformin.^19^ Between 2015 and 2021, NICE technology appraisals and clinical guidelines recommended that, for most people with T2DM, several second‐line oral treatment options should be available, including sodium‐glucose cotransporter 2 (SGLT2) inhibitors, dipeptidyl peptidase‐4 (DPP‐4) inhibitors or the lower‐cost option of sulphonylureas (SUs).^19^, ^20^ Updated NICE guidelines (2022) recommend SGLT2 inhibitors for individuals at high risk of or with prevalent CVD but that, for other eligible patients, any of these three treatments may be suitable.^21^ The decision to allow local discretion in the choice of second‐line treatment may reflect the uncertainty over comparative effectiveness and cost‐effectiveness of these three treatment choices, which is partly related to the lack of randomized controlled trials (RCTs) providing head‐to‐head comparisons of these three oral antidiabetic drugs. In contrast, international diabetes guidance and consensus reports recommend SGLT2 inhibitors for people with established atherosclerotic CVD, heart failure and CKD,^22^ irrespective of the additional costs of SGLT2 inhibitors compared to SUs, drawing on evidence from placebo‐controlled RCTs showing improved CVD and kidney disease outcomes when prescribing SGLT2 inhibitors.

Previous research has found wide variation in clinical practice in the United Kingdom (UK) in the choice of second‐line oral antidiabetic treatment.^23^, ^24^ However, no previous study has considered whether disparities exist in prescriptions of this second‐line treatment according to ethnicity and socioeconomic status. We aimed to assess whether ethnic minorities and people with higher deprivation status had a lower probability of being prescribed SGLT2 inhibitors compared with DPP‐4 inhibitors or the lower‐cost SUs, both overall and by prevalent CVD status.

## 2 METHODS

### 2.1 Study design and setting

We conducted a cross‐sectional study, nested within the Personalized Medicine for Intensification of Treatment in people with T2DM (PERMIT) cohort study,^25^ to investigate disparities in second‐line antidiabetic treatment prescribed to people with T2DM by ethnicity and by deprivation status. Data sources included the Clinical Practice Research Datalink (CPRD) Gold and Aurum datasets (primary care), Hospital Episode Statistics (HES; secondary care), the Index of Multiple Deprivation (IMD), and the Office of National Statistics (ONS) death data.

The CPRD is a large, population‐based dataset covering approximately 20% of the UK population and is representative in terms of age, sex and ethnicity.^26^, ^27^ These data include clinical diagnoses, laboratory test results, and prescribing information recorded in primary care. Linkage of CPRD data to HES data is available for approximately 80% of people in the CPRD registered at general practices in England. HES data include diagnoses and demographic information related to all NHS‐funded hospitalizations.^28^ The IMD is commonly used in epidemiological research as a proxy for socioeconomic status in England. It ranks individuals according to deprivation status based on their postcode, and is usually reported in quintiles (1 being the least deprived, 5 being most deprived).^29^ ONS mortality data include information on all deaths registered in England and Wales.^30^

### 2.2 Patient and public involvement

One patient and public (PP) representative (P.C.) was involved in this study's design, provided feedback on this manuscript, and is a co‐author. The PERMIT study protocol describes PP contributions to the study design.^25^ PP representatives will assist with drafting lay summaries, which we will share on the study website (https://www.lshtm.ac.uk/research/centres-projects-groups/permit) and at study workshops with a wider group of multi‐ethnic PP representatives. We will work with the Centre for Ethnic Health Research, led by co‐author K.K., to make culturally adapted lay summaries.

### 2.3 Study population

We included people aged 18 years or older with a T2DM diagnosis, in whom incident second‐line oral antidiabetic treatment was prescribed for the first time between January 1, 2014 and March 31, 2020 after first‐line antidiabetic treatment with metformin monotherapy. We used the complete historical general practice (GP) electronic health record to ensure this was the first time each person had a record of being prescribed an SU, a DPP‐4 inhibitor or an SGLT2 inhibitor. The second‐line therapy, an SU, a DPP‐4 inhibitor or an SGLT2 inhibitor, had to have been added on to metformin, and had not replaced it. These three treatments constituted approximately 99% of the second‐line treatments prescribed, therefore, other second‐line antidiabetic treatments were excluded from this study.^23^, ^31^ Eligible people had to have had a prescription for metformin monotherapy within 60 days prior to the first prescription for second‐line treatment to ensure they were continuous users of metformin monotherapy prior to intensification. Also, to ensure the second‐line treatments were an addition to, rather than a switch from, metformin, the individuals were required to have been prescribed metformin on the same day or within 60 days after the first prescription for the second‐line antidiabetic treatment.

We excluded women with a record of pregnancy within 12 months prior to second‐line treatment initiation since antidiabetic prescribing guidelines are different for this population.^19^ We also excluded people whose last recorded estimated glomerular filtration rate (eGFR) was less than 30 ml/min/1.73 m^2^ since metformin is contraindicated in this group, and SGLT2 inhibitors are not recommended for this group in the United Kingdom for the purpose of lowering blood glucose.^19^, ^32^

### 2.4 Definitions of ethnicity and deprivation

We defined ethnicity according to clinical and demographic codes recorded within the CPRD or linked HES data prior to or on the same day as the first‐ever prescription date for one of the three second‐line antidiabetic treatments of interest, that is, the index date. Ethnicity was grouped into 16 categories in primary care and 11 categories in secondary care, which we further re‐grouped as the following: (1) White, (2) South Asian, (3) Black, and (4) Mixed/other (Table S1). We considered this re‐grouping necessary to ensure sufficient sample sizes within each ethnic group, and to follow precedent studies using the same data sources,^12^, ^33^, ^34^ as well as the ethnic groupings used in the 2011 England and Wales census.^35^ If the two sources for ethnicity provided different categorizations, then we used ethnicity as defined in the CPRD since these data have been shown to be more reliable than HES inpatient data.^33^ If no ethnicity data were available within the CRPD, we categorized ethnicity using HES data. If ethnicity was not recorded in either source, we considered ethnicity as missing and the individual was excluded from the complete case analyses.

We used the small area IMD to define deprivation. The IMD combines seven indices which capture dimensions of deprivation at the Lower‐Layer Super Output Area or neighbourhood level, and ranks each neighbourhood from 1 to 32 844.^29^ Neighbourhood rankings were divided into quintiles and used to compare relative levels of deprivation among people in this study living in different neighbourhoods in England.

We also considered how the proportion of patients receiving the alternative second‐line treatments may differ according to calendar time, recognizing that the dissemination and awareness of the safety and efficacy of SGLT2 inhibitors for patients with T2DM increased over the time period, with the publication of important RCT results.^36^, ^37^, ^38^ We considered this hypothesis in grouping calendar time into years 2014, 2015 to 2016, 2017 to 2018, and 2019 to 2020.

### 2.5 Covariates

We adjusted for several additional variables, derived from data captured before or on the same day as the index date. These were sex, age, duration of time on metformin monotherapy, number of patients registered at the individual's general practice, geographic region, co‐prescriptions for renin‐angiotensin system inhibitors and/or statins, history of proteinuria, history of hypoglycaemia, clinical measures (body mass index [BMI] and HbA1c), smoking status, alcohol intake, and comorbidities at the time of second‐line antidiabetic treatment initiation. Comorbidities included CKD stage (no known CKD, stages 1, 2, 3a, and 3b, assigned using the latest recorded eGFR), cancer (any), blindness, congestive heart failure, previous myocardial infarction (MI), unstable angina, previous stroke, other ischaemic heart disease, and uncontrolled hypertension based on the most recent blood pressure measures recorded in primary care. We defined prevalent CVD as a composite of heart failure, ischaemic heart disease, unstable angina, previous myocardial infarction, or previous stroke.

### 2.6 Treatment prescribed

Our dependent variable of interest was incident second‐line oral antidiabetic treatment prescribed (SUs, DPP‐4 inhibitors, or SGLT2 inhibitors, in addition to metformin), defined using CPRD prescribing data.

### 2.7 Analysis

We described baseline characteristics of the study population stratified by ethnicity and IMD. We then built mixed‐effect multivariable, multinomial logistic regression models which compared the odds of initiating SGLT2 inhibitors and DPP‐4 inhibitors versus SUs (reference outcome), as well as, in a separate model, SGLT2 inhibitors versus DPP‐4 inhibitors (reference outcome), first adjusting for just age and sex. In the final adjusted model, we adjusted for all covariates, as well as mutual adjustment for ethnicity and deprivation (fixed effects) and clustering at the Clinical Commissioning Group (CCG) level (random effect). Because odds ratios can be misleading, particularly when the outcome is common,^39^ we calculated and plotted predicted percentages from the adjusted model using recycled predictions.^40^ These percentages refer to people prescribed each second‐line antidiabetic treatment stratified by ethnicity, and separately by deprivation, while still adjusting for all measured covariates, and accounting for clustering at the CCG level. We obtained *P* values from Wald tests comparing the predicted percentage of being prescribed one of SUs, DPP‐4 inhibitors or SGLT2 inhibitors by each non‐White ethnic group versus White ethnic group and by deprivation Quintiles 2 to 5 versus deprivation Quintile 1. We also performed joint tests to test whether predicted percentages for each ethnic group or for each deprivation quintile were equal for each second‐line antidiabetic treatment. These percentages and *P* values were used to support our final conclusions on disparities in second‐line antidiabetic treatment prescribing by ethnicity or by deprivation.^39^ We then stratified the adjusted predicted percentages by prevalent CVD status at baseline to determine if there were differences in prescribing by ethnicity or by deprivation quintile according to prevalent CVD status.

In the secondary analyses, we compared the change in odds ratios between the fixed‐effect model (model including ethnicity, deprivation, and all covariates) and the mixed‐effect model (the fixed effect model plus accounting for CCG clustering as a random effect). We also compared the final adjusted mixed‐effect model with and without adjustment for deprivation to observe any changes in ethnic disparities in second‐line antidiabetic treatment prescribed when adding this variable to the multivariable model, since deprivation could be a mediator between ethnicity and second‐line antidiabetic treatment prescribed. Because awareness of the cardio‐ and kidney‐protective effects of SGLT2 inhibitors versus placebo have increased over time, we considered year of second‐line oral antidiabetic treatment initiation as the independent variable of interest, repeating the main analysis to observe any differences in second‐line antidiabetic treatment prescribed over time, overall and stratified by prevalent CVD status. Finally, we investigated whether there were interactions between (1) ethnicity and IMD, (2) ethnicity and calendar time, and (3) deprivation and calendar time, informed by the results of likelihood ratio tests on the final adjusted multinomial models, and by joint tests on whether predicted percentages for interaction terms were equal for each of the three second‐line treatments prescribed.

Data management and analyses were performed using Stata 17.

## 3 RESULTS

### 3.1 Baseline characteristics

**FIGURE 1:**
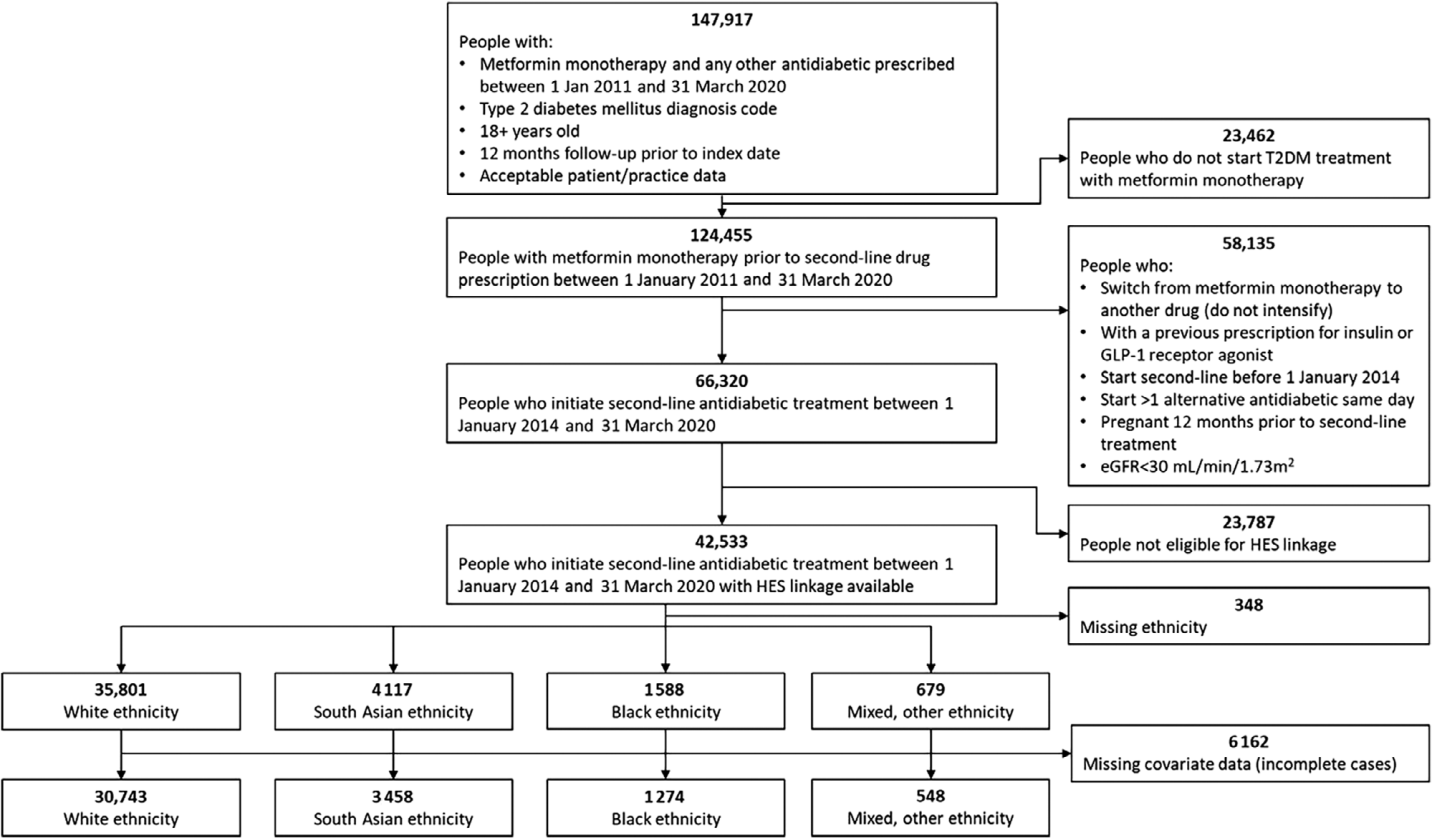
Flow diagram illustrating selection of study population diagnosed with type 2 diabetes mellitus (T2DM) and initiating second‐line antidiabetic treatment. eGFR, estimated glomerular filtration rate; HES, Hospital Episodes Statistics.

The study population included 36 023 people with complete data on all variables of interest who initiated second‐line oral antidiabetic treatment during the study period with linked secondary care data (Figure 1). Eighty‐four percent of the cohort were White, 10% were South Asian, 4% were Black, and 1% were Mixed/other ethnicity. We excluded 6150 people with missing data for at least one variable, including 348 with missing ethnicity data and 20 with missing IMD data (Table S4).

Overall, 41% of the cohort was female, with a mean age of 59 years (Table 1). People of White ethnicity were more likely to be male (60%) and older (mean age 60.1 years) compared with people of South Asian ethnicity (53% male, mean age 52.6 years), Black ethnicity (49% male, mean age 55.2 years), and people of Mixed/other ethnicity (58% male, mean age 55.4 years). People of South Asian and Black ethnicities were over‐represented in the lowest IMD quintile (34% and 46%, respectively). Recorded CVD prevalence was 24% overall and was lower in people of Black (15%) compared to White ethnicity (23%). Mean BMI was highest in people of White ethnicity (33.8 kg/m^2^) and lowest in people with South Asian ethnicity (30.2 kg/m^2^).

**TABLE 1:** Baseline characteristics of study population (at time of second‐line treatment initiation)

	Total	White	South Asian	Black	Mixed/Other
	36 023 (100%)	30 743 (85%)	3458 (10%)	1274 (4%)	548 (1%)
**Female, n (%)**	14 643 (41)	12 149 (40)	1616 (47)	646 (51)	232 (42)
**Age at baseline, n (%)**					
18‐49 years	7734 (21)	5711 (19)	1450 (42)	398 (31)	175 (32)
50–59 years	11 128 (31)	9396 (31)	1059 (31)	483 (38)	190 (35)
60‐69 years	9622 (27)	8613 (28)	662 (19)	236 (19)	111 (20)
70+ years	7539 (21)	7023 (23)	287 (8)	157 (12)	72 (13)
**Year of second‐line treatment initiation, n (%)**					
2014	4092 (11)	3584 (12)	318 (9)	139 (11)	51 (9)
2015‐2016	10 910 (30)	9467 (31)	915 (26)	364 (29)	164 (30)
2017‐2018	12 221 (34)	10 379 (34)	1186 (34)	459 (36)	197 (36)
2019‐2020	8800 (24)	7313 (24)	1039 (30)	312 (24)	136 (25)
**Years on first‐line (metformin monotherapy), n (%)**					
<0.5	6676 (19)	5710 (19)	539 (16)	320 (25)	107 (20)
0.5‐0.99	3654 (10)	3183 (10)	288 (8)	123 (10)	60 (11)
≥1	25 693 (71)	21 850 (71)	2631 (76)	831 (65)	381 (70)
**Median (IQR) no. of patients registered at the person's GP**	10 295 (6981‐14254)	10 471 (7184‐14275)	8793 (5213‐12861)	10 357 (6484‐15253)	10 674 (7377‐15196)
**Hospitalization (any) within 1 year prior to second‐line treatment initiation, n (%)**	10 216 (28)	8818 (29)	873 (25)	361 (28)	164 (30)
**IMD quintile, n (%)**					
1 (least deprived)	5739 (16)	5322 (17)	271 (8)	45 (4)	101 (18)
2	6484 (18)	5922 (19)	419 (12)	77 (6)	66 (12)
3	6915 (19)	6075 (20)	601 (17)	153 (12)	86 (16)
4	8020 (22)	6473 (21)	995 (29)	416 (33)	136 (25)
5 (most deprived)	8865 (25)	6951 (23)	1172 (34)	583 (46)	159 (29)
**HbA1c at baseline, n (%)**		9.1	8	9	9.1
<53 mmol/mol (7%)	1318 (4)	1070 (3)	139 (4)	84 (7)	25 (5)
53‐74 mmol/mol	19 443 (54)	16 548 (54)	2057 (59)	541 (42)	297 (54)
75+ mmol/mol (9%)	15 262 (42)	13 125 (43)	1262 (36)	649 (51)	226 (41)
**Uncontrolled hypertension, based on last recorded blood pressure, n (%)**					
Normotensive	9749 (27)	8073 (26)	1155 (33)	351 (28)	170 (31)
Hypertensive	26 274 (73)	22 670 (74)	2303 (67)	923 (72)	378 (69)
**Mean (SD) BMI, kg/m** ^**2**^	33.4 (7.0)	33.8 (7.1)	30.2 (5.8)	32.2 (6.9)	31.3 (6.8)
**Smoking status, n (%)**					
Non‐smoker	7371 (20)	5691 (19)	1145 (33)	389 (31)	146 (27)
Current smoker	9874 (27)	8608 (28)	804 (23)	317 (25)	145 (26)
Ex‐smoker	18 778 (52)	16 444 (53)	1509 (44)	568 (45)	257 (47)
**Alcohol status, n (%)**					
Non‐drinker	3846 (11)	2276 (7)	1199 (35)	239 (19)	132 (24)
Current drinker	22 082 (61)	20 136 (65)	1112 (32)	583 (46)	251 (46)
Ex‐drinker	10 095 (28)	8331 (27)	1147 (33)	452 (35)	165 (30)
**Co‐prescriptions, n (%)**					
RAS inhibitors	17 949 (50)	15 700 (51)	1450 (42)	535 (42)	264 (48)
Statins	24 907 (69)	21 472 (70)	2376 (69)	713 (56)	346 (63)
**Cancer (any)**	4048 (11)	3794 (12)	129 (4)	91 (7)	34 (6)
**Macrovascular comorbidities, n (%)**					
CVD composite^a^	8466 (24)	7589 (25)	600 (17)	191 (15)	86 (16)
Amputation	283 (1)	270 (1)	8 (0)	<5(0)	<5 (0)
Heart failure	2110 (6)	1936 (6)	109 (3)	51 (4)	14 (3)
Myocardial infarction	2521 (7)	2288 (7)	174 (5)	37 (3)	22 (4)
Stroke	1640 (5)	1470 (5)	103 (3)	54 (4)	13 (2)
Ischaemic heart disease	6823 (19)	6117 (20)	495 (14)	134 (11)	77 (14)
Unstable angina	1175 (3)	1049 (3)	83 (2)	29 (2)	14 (3)
**Microvascular comorbidities, n (%)**					
**eGFR at baseline category (mL/min/1.73 m^2^)**					
No known CKD (eGFR missing)	675 (2)	612 (2)	31 (1)	11 (1)	21 (4)
90+ (Stage 1)	21 391 (59)	17 676 (57)	2694 (78)	640 (50)	381 (70)
60‐89 (Stage 2)	11 913 (33)	10 608 (35)	648 (19)	539 (42)	118 (22)
45‐59 (Stage 3a)	1585 (4)	1432 (5)	65 (2)	66 (5)	22 (4)
30‐44 (Stage 3b)	459 (1)	415 (1)	20 (1)	18 (1)	6 (1)
Blindness	486 (1)	432 (1)	33 (1)	13 (1)	8 (1)
Hypoglycaemia	320 (1)	272 (1)	23 (1)	16 (1)	9 (2)
Proteinuria	2586 (7)	2202 (7)	260 (8)	80 (6)	44 (8)

^a^ CVD composite: heart failure, ischaemic heart disease, myocardial infarction, stroke, unstable angina.

Abbreviations: BMI, body mass index; CPRD, Clinical Practice Research Datalink; CVD, cardiovascular; DPP‐4, dipeptidyl peptidase‐4; eGFR, estimated glomerular filtration rate; GP, general practice; HES, Hospital Episode Statistics; IMD, Index of Multiple Deprivation; IQR, interquartile range; RAS, renin‐angiotensin system; SGLT2, sodium‐glucose cotransporter 2; SD, standard deviation; SU, sulphonylureas; UK, United Kingdom.

After stratifying by IMD quintile, we found that people in the most deprived quintile were over‐represented (25%) and people in the least deprived quintile were under‐represented (16%; Table S2). People in the most deprived quintile were younger (mean age 56.6 years) compared with the least deprived quintile (mean age 61.8 years). The most deprived quintile included a higher proportion of South Asian and Black people (13% and 7%, respectively) compared with the least deprived quintile (5% and 1%, respectively).

People with missing covariate information were similar according to sex, age, IMD and prevalent CVD versus those with fully observed covariate information (Tables S3,S4).

### 3.2 Ethnicity and second‐line antidiabetic treatment choice

In people of Black ethnicity, the most common second‐line treatment during the study period was SUs (593 [46%]), whereas for all other ethnic groups DPP‐4 inhibitors was the most commonly prescribed second‐line treatment (13 398 [43%], 1530 [44%], and 249 [45%], among people of White, South Asian, and Mixed/other ethnic groups, respectively; Table 2). SGLT2 inhibitors were the least common second‐line treatment across all ethnic groups, ranging from 14% prescribed, among people of Black ethnicity, to 21%, among people of White ethnicity (Table 2).

**TABLE 2:** Crude proportions of second‐line treatment prescribed by ethnicity or by deprivation

	Second‐line antidiabetic treatment prescribed		
Variable of interest	Metformin‐SUs	Metformin‐DPP‐4 inhibitors	Metformin‐SGLT2 inhibitors
Ethnicity, *n* (row %)			
White	11 584 (37)	13 398 (43)	6455 (21)
South Asian	1316 (37)	1530 (44)	667 (19)
Black	593 (46)	524 (40)	179 (14)
Mixed/other	216 (39)	249 (45)	93 (17)
IMD quintile, *n* (row %)			
1 (least deprived)	2155 (37)	2483 (42)	1264 (21)
2	2432 (37)	2833 (43)	1388 (21)
3	2654 (37)	2972 (42)	1476 (21)
4	3112 (38)	3975 (44)	1628 (20)
5 (most deprived)	3356 (37)	3975 (44)	1638 (18)

Abbreviations: DPP‐4, dipeptidyl peptidase‐4; IMD, Index of Multiple Deprivation; SGLT2, sodium‐glucose cotransporter 2; SU, sulphonylurea.

**FIGURE 2:**
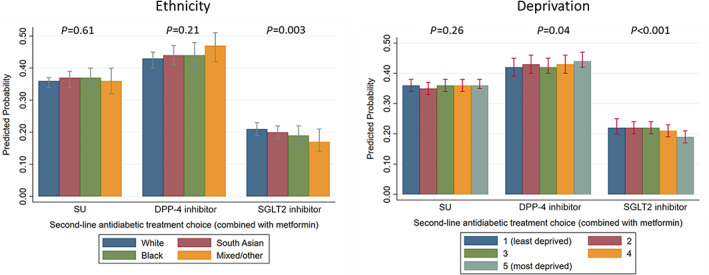
Adjusted predicted percentages of second‐line antidiabetic treatment prescribed, according to ethnicity or deprivation. *P* values are generated from joint tests comparing the adjusted predicted percentages for being prescribed a particular second‐line antidiabetic drug (a sulphonylurea [SU], a dipeptidyl peptidase‐4 [DPP‐4] inhibitor or a sodium‐glucose cotransporter‐2 [SGLT2] inhibitor) across ethnic groups or across deprivation levels. Predicted percentages are mutually adjusted for deprivation (ethnicity estimates) and ethnicity (deprivation estimates), as well as the number of patients registered at the patient's general practice, years on first‐line treatment category, age category, sex, last glycated haemoglobin value prior to second‐line treatment initiation category, body mass index, prevalent heart failure, ischaemic heart disease, myocardial infarction, stroke, unstable angina, renin‐angiotensin system inhibitors and/or statin co‐prescription, chronic kidney disease category, blood pressure category, history of proteinuria, blindness, cancer (any), hospitalization (any) in past year, smoking status, alcohol status, region, all as fixed effects, and Clinical Commissioning Group‐clustering as a random effect.

There was some evidence that adjusted predicted percentages for being prescribed SGLT2 inhibitors were greatest for White people (21% [95% CI 19–23%]) compared with South Asian people (20% [95% CI 18–22%]), Black people (19% [95% CI 16–22%]) and Mixed/other people (17% [95% CI 14–21%]; *P* = 0.003 [Figure 2, Table S5]). There was no evidence of differences in adjusted predicted percentages for being prescribed DPP‐4 inhibitors or SUs according to ethnicity (Figure 2, Table S5). The results from the multinomial, multivariable logistic regression model used to calculate these adjusted predicted percentages are described in Table S6.

### 3.3 Social deprivation and second‐line antidiabetic treatment choice

The crude proportion of people prescribed each second‐line antidiabetic treatment option across deprivation quintiles are presented in Table 2.

There was some evidence of a small difference in adjusted predicted percentages of people prescribed SGLT2 inhibitors according to deprivation: 19% (95% CI 17–21%) were prescribed SGLT2 inhibitors in the most deprived quintile, and 22% (95% CI 20–24%) were prescribed SGLT2 inhibitors in the least deprived quintile (*P* < 0.001; Figure 2, Table S5). Conversely, there was some evidence that people in the most deprived quintile had a small increase in the adjusted predicted percent of being prescribed DPP‐4 inhibitors 44% (95% CI 42–47%) versus 42% (95% CI 39–45%) of people in the least deprived quintile (*P* = 0.04). There was no evidence of any differences in the adjusted predicted percentages of people prescribed SUs according to deprivation (*P* = 0.26).

### 3.4 Second‐line antidiabetic treatment prescribed among people with prevalent CVD

When stratifying by prevalent CVD status (*n* = 8466 with prevalent CVD, *n* = 27 557 without prevalent CVD), adjusted predicted percentages showed no substantial differences in SU prescribing across ethnicities and across deprivation quintiles. However, adjusted predicted percentages showed evidence of a slightly higher proportion of people prescribed DPP‐4 inhibitors with prevalent CVD versus no CVD across all ethnicities. Conversely, adjusted predicted percentages showed evidence of slightly less SGLT2 inhibitor prescribing among people with prevalent CVD versus no CVD across all ethnicities (Table S7).

### 3.5 Secondary analyses

Results were similar in the fixed‐effect and mixed‐effect models (Table S6), and we did not observe any substantial mediation by deprivation level on the association between ethnicity and second‐line treatment prescribed (Table S8).

Second‐line antidiabetic treatments prescribed changed over time (Tables S9–S11, Figure S1). Briefly, SU prescribing decreased substantially over time (adjusted predicted percent 60% [95% CI 57–62%] in 2014 versus 23% [95% CI 21–24%] in 2019–2020), while prescribing of DPP‐4 inhibitors and SGLT2 inhibitors increased over the same time periods (DPP‐4 inhibitors: 34% [95% CI 31–36%] in 2014 versus 43% [95% CI 40–46%] in 2019–2020; and SGLT2 inhibitors: 7% [95% CI 5–8%] in 2014 versus 34% [95% CI 32–37%] in 2019–2020; Table S10). Among people with and without prevalent CVD, those with prevalent CVD had consistently lower probabilities of being prescribed SGLT2 inhibitors compared with those without prevalent CVD across time periods (Table S10).

There was no evidence of an interaction effect between ethnicity and IMD on second‐line antidiabetic treatment prescribed on the odds scale (*P* = 0.45). On the adjusted predicted percent scale, there was some evidence that the percent prescribed SGLT2 inhibitors among people of White ethnicity and Mixed/other ethnicity decreased with increasing deprivation (*P* < 0.001 and *P* = 0.04). There was no evidence of an interaction between calendar time and ethnicity (*P* = 0.11), nor between calendar time and deprivation quintile (*P* = 0.66).

## 4 DISCUSSION

We found statistically significant, but small absolute differences in SU, DPP‐4 inhibitor and SGLT2 inhibitor prescribing as second‐line antidiabetic treatment, in combination with metformin, according to ethnicity and deprivation level, after accounting for several covariates and clustering at the CCG level in England. There was some evidence that across ethnic groups and levels of deprivation, people with prevalent CVD had a lower probability of being prescribed SGLT2 inhibitors compared with those without prevalent CVD.

It is reassuring that we did not observe substantial ethnic differences in second‐line antidiabetic treatment prescribing, as previous research has described many other ethnic disparities related to T2DM. In the UK, ethnic minorities with T2DM had longer delays in intensification to second‐line treatment than White people with T2DM, and experienced greater treatment inertia following identification of uncontrolled HbA1c.^12^ In the United States, which does not have a universal healthcare system, ethnic minorities are less likely to be prescribed SGLT2 inhibitors at any time after T2DM diagnosis compared to White people, even after adjusting for deprivation level.^41^

This cross‐sectional study used one of the largest primary care datasets in the world.^26^, ^27^ While our results suggest statistical evidence of a lower percentage of ethnic minorities and people from more deprived areas in England being prescribed SGLT2 inhibitors compared with people of White ethnicity, these differences were not substantial and unlikely to represent major disparities in T2DM care. Factors such as willingness to try newer treatments on the part of both the healthcare team and the patient are unmeasured, and could have contributed to the small differences in second‐line antidiabetic treatments prescribed that we observed in this study.

It is, however, concerning that people with prevalent CVD had a lower probability of receiving SGLT2 inhibitors versus those without, since trials comparing SGLT2 inhibitors versus placebo have shown substantial improvements in diabetic‐related outcomes among those with atherosclerotic CVD, heart failure, and kidney disease.^38^, ^42^, ^43^ However, national and international guidance/guidelines recommending SGLT2 inhibitors among those with prevalent CVD were only updated towards the end of our study period.^22^, ^44^ We hope future research shows increased SGLT2 inhibitor prescribing in those with prevalent CVD after 2020.

This study has some limitations. We did not include people with missing documentation of ethnicity; however, only 348 people (0.8%) in the total sample had missing ethnicity data, which limited our ability to include these people as a separate group in our analyses. Further, some exposure misclassification may exist in our study, since deprivation was measured at the small area/neighbourhood level and patient‐level deprivation status may differ from this measure. There is also likely to be residual confounding according to other clinical characteristics such as history of alcohol misuse, pancreatic disease, urinary tract infections, mycotic urinary infections, and unobserved factors such as prescriber characteristics and patient frailty status. Our data came from electronic health records, which are not designed primarily for research and thus some degree of misclassification is expected for covariates, particularly those which are not necessarily recorded on the same day as second‐line treatment initiation (eg, HbA1c, BMI, eGFR, blood pressure). Finally, we were limited to prescribing data from primary care to define second‐line antidiabetic treatment choice. We were unable to use dispensing data from pharmacies since these data are not available for linkage to CPRD data, nor were we able to determine if treatment initiation occurred during an inpatient stay (secondary care), where prescription of newer drug classes may be relatively more likely. We adjusted for any hospitalization in the past year to try and account for this. However, even if a prescription was initiated or recommended by specialist care, primary care would probably continue prescriptions of these therapies.

In conclusion, we found statistically significant, but small differences in second‐line oral antidiabetic treatment prescribing by ethnicity and social deprivation status in England. These differences are unlikely to be clinically important. We consider it encouraging that, after accounting for various clinical characteristics and variation at the CCG level, ethnic minorities and people from more deprived backgrounds did not have substantially lower probabilities of being prescribed SGLT2 inhibitors compared with DPP‐4 inhibitors and SUs. Future work should investigate other factors at the individual and local CCG level which may drive treatment choice to understand how these treatments are used in routine care, and to highlight the need for future research to directly evaluate the comparative effectiveness of these three second‐line antidiabetic treatment choices to optimize oral antidiabetic treatment prescribing.

## AUTHOR CONTRIBUTIONS

Patrick Bidulka, David Lugo‐Palacios, Stephen O'Neill, Anirban Basu, Kamlesh Khunti and Richard Grieve conceived and designed the study. Patrick Bidulka and Stephen O'Neill conducted the data management and analyses. Patrick Bidulka wrote the first draft of the manuscript. All authors (Patrick Bidulka, Rohini Mathur, David Lugo‐Palacios, Stephen O'Neill, Anirban Basu, Richard Silverwood, Paul Charlton, Andrew Briggs, Liam Smeeth, Amanda Adler, Ian Douglas, Kamlesh Khunti and Richard Grieve) reviewed and commented on the manuscript, and approved the final version for submission.

## CONFLICT OF INTERESTS

All authors have completed an ICJME form. Patrick Bidulka, Stephen O'Neill, Anirban Basu, Richard Silverwood and Liam Smeeth have nothing to declare. Kamlesh Khunti has acted as a consultant, speaker or received grants for investigator‐initiated studies for Astra Zeneca, Novartis, Novo Nordisk, Sanofi‐Aventis, Lilly and Merck Sharp & Dohme, Boehringer Ingelheim, Bayer, Berlin‐Chemie AG / Menarini Group, Janssen and Napp. Rohini Mathur has received consulting fees from AMGEN. Paul Charlton sat on an NIHR HTA Commissioning Committee member until September 2021. Anirban Basu is an economic advisor on the DiRECT trial, with ongoing responsibility for economic analysis during the long‐term follow‐up phase, and has also acted as consultant to GlaxoSmithKline, Merck, Novo Nordisk and Boehringer Ingelheim in relation to their diabetes products. Amanda Adler receives salary from the NIHR BRC via the Oxford Centre for Diabetes, Endocrinology and Metabolism. Ian Douglas holds an unrestricted research grant from GSK and holds shares in GSK. Richard Grieve sits on the NIHR commissioning committee.

### PEER REVIEW

The peer review history for this article is available at https://publons.com/publon/10.1111/dom.14874.

## ETHICS APPROVAL

This research was approved by the London School of Hygiene & Tropical Medicine ethics committee (reference 21 395) and the Independent Scientific Advisory Committee (reference 20_064).

## TRANSPARENCY STATEMENT

Patrick Bidulka, as corresponding author, confirms that the manuscript is an honest, accurate, and transparent account of the study being reported and that no important aspects of the study have been omitted, and that any discrepancies from the study as planned have been explained.

## Supporting information


**Appendix S1:** Supporting information

## Data Availability

Due to data‐sharing restrictions, we cannot share the data used in this study directly. However, researchers may apply to use CPRD data linked with other health datasets. Please see the CPRD website for further instruction https://cprd.com/. Codelists to create exposure, outcome, and covariates will be published on LSHTM DataCompass https://datacompass.lshtm.ac.uk/.
