# Supplementary material for: Ethnic and socioeconomic disparities in initiation of second‐line antidiabetic treatment for people with type 2 diabetes in England: A cross‐sectional study
Source: Diabetes Obes Metab. 2022 Nov 2;25(1):282–92. doi: 10.1111/dom.14874 (PMC10092566; doi:10.1111/dom.14874)

**Supplementary materials**

**Supplementary table 1:** Ethnicity groupings from CPRD and HES, and in this study.

**Supplementary table 2:** Baseline characteristics of CPRD-HES-linked study population stratified by IMD quintile

**Supplementary table 3:** Characteristics of people excluded from the complete case analysis (at least one covariate with missing data) stratified by ethnicity.

**Supplementary table 4:** Summary of missing covariate data in people excluded from the main complete case analysis.

**Supplementary table 5:** Adjusted predicted percentages of second-line treatment prescribed by ethnicity and IMD.

**Supplementary table 6:** Results from the multinomial, multivariable logistic regression model used to calculate adjusted predicted percentages for the association between ethnicity, deprivation, and second-line antidiabetic treatment prescribed

**Supplementary table 7:** Adjusted predicted percentages of second-line treatment choice by ethnicity and IMD, stratified by prevalent CVD status.

**Supplementary table 8:** Describing the potential mediating effect of deprivation on the association between ethnicity and second-line antidiabetic treatment prescribed using results from multivariable, multinomial logistic regression models

**Supplementary table 9:** Second-line antidiabetic treatment prescribed stratified by time, crude proportions, n (row %)

**Supplementary table 10:** Adjusted predicted percentages of second-line treatment prescribed by year of second-line treatment initiation, overall and stratified by prevalent CVD status.

**Supplementary table 11:** Results from the multinomial, multivariable logistic regression model used to calculate adjusted predicted percentages for the association between year of second-line treatment initiation and second-line antidiabetic treatment prescribed

**Supplementary figure 1:** Proportion of people prescribed each second-line antidiabetic between 2014-2020, overall and stratified by prevalent CVD at baseline status.

**Supplementary table 1:** Ethnicity groupings from CPRD and HES, and in this study.

| **CPRD collapsed groupings** | **CPRD ethnicity categories** |
| --- | --- |
| White | British |
|  | Irish |
|  | Other white |
| South Asian | Indian |
|  | Pakistani |
|  | Bangladeshi |
|  | Other Asian |
| Black | Caribbean |
|  | African |
|  | Other black |
| Mixed | White and Black Caribbean |
|  | White and Black African |
|  | White and Asian |
|  | Other mixed |
| Other | Chinese |
|  | Other ethnic group |
| **HES collapsed groupings** | **HES ethnicity categories** |
| White | White |
| South Asian | Bangladeshi |
|  | Indian |
|  | Other Asian |
|  | Pakistani |
| Black | Black African |
|  | Black Caribbean |
|  | Black other |
| Mixed | Mixed |
| Other | Chinese |
|  | Other |

**Supplementary table 2:** Baseline characteristics of CPRD-HES-linked study population stratified by IMD quintile

|  | **Total** | **1 (least deprived)** | **2** | **3** | **4** | **5 (most deprived)** |
| --- | --- | --- | --- | --- | --- | --- |
|  | **36,023 (100%)** | **5,739 (16%)** | **6,484 (18%)** | **6,915 (19%)** | **8,020 (22%)** | **8,865 (25%)** |
| **Female** | 14,643 (41) | 2,158 (38) | 2,428 (37) | 2,736 (40) | 3,399 (42) | 3,922 (44) |
| **Age at baseline in years, mean (SD)** | 59.2 (12.2) | 61.8 (11.9) | 61.0 (12.2) | 59.9 (12.1) | 58.2 (12.0) | 56.5 (11.8) |
| **Age at baseline in years** |  |  |  |  |  |  |
| 18-49 | 7,734 (21) | 842 (15) | 1,137 (18) | 1,349 (20) | 1,907 (24) | 2,499 (28) |
| 50-59 | 11,128 (31) | 1,699 (30) | 1,850 (29) | 2,110 (31) | 2,544 (32) | 2,925 (33) |
| 60-69 | 9,622 (27) | 1,590 (28) | 1,806 (28) | 1,883 (27) | 2,135 (27) | 2,208 (25) |
| 70+ | 7,539 (21) | 1,608 (28) | 1,691 (26) | 1,573 (23) | 1,434 (18) | 1,233 (14) |
| **Year of second-line treatment initiation** |  |  |  |  |  |  |
| 2014 | 4,092 (11) | 650 (11) | 767 (12) | 818 (12) | 876 (11) | 981 (11) |
| 2015-2016 | 10,910 (30) | 1,716 (30) | 1,927 (30) | 2,083 (30) | 2,510 (31) | 2,674 (30) |
| 2017-2018 | 12,221 (34) | 1,951 (34) | 2,152 (33) | 2,352 (34) | 2,660 (33) | 3,106 (35) |
| 2019-2020 | 8,800 (24) | 1,422 (25) | 1,638 (25) | 1,662 (24) | 1,974 (25) | 2,104 (24) |
| **Months on first-line (MTF monotherapy), median (IQR)** | 25 (10-45) | 26 (10-46) | 26 (11-45) | 25 (10-45) | 25 (10-44) | 24 (9-44) |
| **Years on first-line (MTF monotherapy)** |  |  |  |  |  |  |
| <0.5 | 6,676 (19) | 1,062 (19) | 1,184 (18) | 1,243 (18) | 1,497 (19) | 1,690 (19) |
| 0.5-0.99 | 3,654 (10) | 534 (9) | 615 (9) | 706 (10) | 837 (10) | 962 (11) |
| ≥1 | 25,693 (71) | 4,143 (72) | 4,685 (72) | 4,966 (72) | 5,686 (71) | 6,213 (70) |
| **No. of patients registered at the person’s GP, median no. patients (IQR)** | 10295 (6981-14254) | 11605 (8161-15620) | 10798 (7395-14695) | 10451 (6833-14279) | 10295 (6954-14379) | 9011 (6083-13130) |
| **No. of patients registered at the person’s GP** |  |  |  |  |  |  |
| <7,000 | 9,090 (25) | 1,053 (18) | 1,435 (22) | 1,784 (26) | 2,031 (25) | 2,787 (31) |
| 7,000-10,000 | 7,975 (22) | 1,167 (20) | 1,488 (23) | 1,436 (21) | 1,729 (22) | 2,155 (24) |
| 10,000-15,000 | 11,157 (31) | 1,966 (34) | 2,004 (31) | 2,207 (32) | 2,536 (32) | 2,444 (28) |
| >=15,000 | 7,801 (22) | 1,553 (27) | 1,557 (24) | 1,488 (22) | 1,724 (21) | 1,479 (17) |
| **Hospitalisation (any) within 1 year prior to second-line treatment initiation** | 10,216 (28) | 1,587 (28) | 1,753 (27) | 1,965 (28) | 2,279 (28) | 2,632 (30) |
| **Ethnicity** |  |  |  |  |  |  |
| White | 30,743 (85) | 5,322 (93) | 5,922 (91) | 6,075 (88) | 6,473 (81) | 6,951 (78) |
| South Asian | 3,458 (10) | 271 (5) | 419 (6) | 601 (9) | 995 (12) | 1,172 (13) |
| Black | 1,274 (4) | 45 (1) | 77 (1) | 153 (2) | 416 (5) | 583 (7) |
| Mixed/other | 548 (2) | 101 (2) | 66 (1) | 86 (1) | 136 (2) | 159 (2) |
| **HbA1c at baseline (mmol/mol), mean (SD)** | 76 (20) | 76 (19) | 76 (20) | 76 (20) | 76 (20) | 77 (21) |
| **HbA1c at baseline (mmol/mol)** |  |  |  |  |  |  |
| <53 (7%) | 1,318 (4) | 191 (3) | 249 (4) | 240 (3) | 307 (4) | 331 (4) |
| 53-74 | 19,443 (54) | 3,258 (57) | 3,535 (55) | 3,754 (54) | 4,321 (54) | 4,575 (52) |
| 75+ (9%) | 15,262 (42) | 2,290 (40) | 2,700 (42) | 2,921 (42) | 3,392 (42) | 3,959 (45) |
| **Blood pressure (mm Hg)** |  |  |  |  |  |  |
| Systolic, mean (SD) | 132 (14) | 132 (13) | 132 (14) | 132 (14) | 131 (14) | 131 (14) |
| Diastolic, mean (SD) | 78 (9) | 78 (9) | 78 (9) | 78 (9) | 78 (9) | 79 (9) |
| **Uncontrolled hypertension, based on most recent blood pressure measure** |  |  |  |  |  |  |
| Normotensive | 9,749 (27) | 1,547 (27) | 1,700 (26) | 1,770 (26) | 2,223 (28) | 2,509 (28) |
| Hypertensive | 26,274 (73) | 4,192 (73) | 4,784 (74) | 5,145 (74) | 5,797 (72) | 6,356 (72) |
| **BMI (kg/m^2^), mean (SD)** | 33.4 (7.0) | 32.4 (6.5) | 33.1 (6.9) | 33.3 (7.0) | 33.6 (7.2) | 34.1 (7.3) |
| **BMI category** |  |  |  |  |  |  |
| Under/normal weight | 2,962 (8) | 579 (10) | 549 (8) | 562 (8) | 653 (8) | 619 (7) |
| Overweight | 9,436 (26) | 1,700 (30) | 1,776 (27) | 1,827 (26) | 1,992 (25) | 2,141 (24) |
| Obese | 23,625 (66) | 3,460 (60) | 4,159 (64) | 4,526 (65) | 5,375 (67) | 6,105 (69) |
| **Smoking status** |  |  |  |  |  |  |
| Non-smoker | 7,371 (20) | 1,398 (24) | 1,428 (22) | 1,458 (21) | 1,557 (19) | 1,530 (17) |
| Current smoker | 9,874 (27) | 1,102 (19) | 1,501 (23) | 1,778 (26) | 2,368 (30) | 3,125 (35) |
| Ex-smoker | 18,778 (52) | 3,239 (56) | 3,555 (55) | 3,679 (53) | 4,095 (51) | 4,210 (47) |
| **Alcohol status** |  |  |  |  |  |  |
| Non-drinker | 3,846 (11) | 428 (7) | 530 (8) | 740 (11) | 981 (12) | 1,167 (13) |
| Current drinker | 22,082 (61) | 4,058 (71) | 4,393 (68) | 4,348 (63) | 4,595 (57) | 4,688 (53) |
| Ex-drinker | 10,095 (28) | 1,253 (22) | 1,561 (24) | 1,827 (26) | 2,444 (30) | 3,010 (34) |
| **Co-prescriptions** |  |  |  |  |  |  |
| RASi | 17,949 (50) | 2,901 (51) | 3,349 (52) | 3,473 (50) | 3,915 (49) | 4,311 (49) |
| Statins | 24,907 (69) | 3,809 (66) | 4,377 (68) | 4,731 (68) | 5,608 (70) | 6,382 (72) |
| **Macrovascular comorbidities** |  |  |  |  |  |  |
| CVD composite* | 8,466 (24) | 1,296 (23) | 1,462 (23) | 1,653 (24) | 1,874 (23) | 2,181 (25) |
| Amputation | 283 (1) | 29 (1) | 55 (1) | 51 (1) | 63 (1) | 85 (1) |
| Heart failure | 2,110 (6) | 316 (6) | 356 (5) | 412 (6) | 485 (6) | 541 (6) |
| Myocardial infarction | 2,521 (7) | 381 (7) | 433 (7) | 510 (7) | 564 (7) | 633 (7) |
| Stroke | 1,640 (5) | 247 (4) | 278 (4) | 304 (4) | 357 (4) | 454 (5) |
| Ischaemic heart disease | 6,823 (19) | 1,039 (18) | 1,170 (18) | 1,343 (19) | 1,510 (19) | 1,761 (20) |
| Unstable angina | 1,175 (3) | 169 (3) | 190 (3) | 226 (3) | 274 (3) | 316 (4) |
| Cancer (any) | 4,048 (11) | 817 (14) | 826 (13) | 828 (12) | 805 (10) | 772 (9) |
| **Microvascular comorbidities** |  |  |  |  |  |  |
| eGFR at baseline (mL/min/1.73m^2^), mean (SD) | 92 (18) | 90 (18) | 90 (18) | 91 (18) | 93 (18) | 94 (18) |
| eGFR at baseline category (mL/min/1.73m^2^) |  |  |  |  |  |  |
| No known CKD (eGFR missing) | 675 (2) | 209 (4) | 187 (3) | 118 (2) | 93 (1) | 68 (1) |
| 90+ (Stage 1) | 21,391 (59) | 3,108 (54) | 3,644 (56) | 4,010 (58) | 4,919 (61) | 5,710 (64) |
| 60-89 (Stage 2) | 11,913 (33) | 2,097 (37) | 2,223 (34) | 2,378 (34) | 2,567 (32) | 2,648 (30) |
| 45-59 (Stage 3a) | 1,585 (4) | 239 (4) | 336 (5) | 321 (5) | 349 (4) | 340 (4) |
| 30-44 (Stage 3b) | 459 (1) | 86 (1) | 94 (1) | 88 (1) | 92 (1) | 99 (1) |
| Blindness | 486 (1) | 56 (1) | 77 (1) | 96 (1) | 125 (2) | 132 (1) |
| Hypoglycaemia | 320 (1) | 53 (1) | 50 (1) | 64 (1) | 63 (1) | 90 (1) |
| Proteinuria | 2,586 (7) | 441 (8) | 430 (7) | 497 (7) | 570 (7) | 648 (7) |
|  |  |  |  |  |  |  |
|  |  | | | | |  |

*CVD composite: heart failure, IHD, MI, stroke, unstable angina

BMI: body mass index; CPRD: Clinical Practice Research Datalink; CVD: cardiovascular; DPP4i: dipeptidyl peptidase-4 inhibitors; eGFR: estimated glomerular filtration rate; GP: general practice; HES: Hospital Episode Statistics; IMD: index of multiple deprivation; IQR: interquartile range; MTF: metformin; RASi: renin-angiotensin system inhibitors; SGTL2i: sodium-glucose co-transporter 2 inhibitors; SD: standard deviation; SU: sulfonylureas; TZD: thiazolidinediones; UK: United Kingdom

**Supplementary table 3:** Characteristics of people excluded from the complete case analysis (at least one covariate with missing data) stratified by ethnicity.

|  | **Total** | **White** | **South Asian** | **Black** | **Mixed/Other** |
| --- | --- | --- | --- | --- | --- |
|  | **6,162 (100%)** | **5,058 (82%)** | **659 (11%)** | **314 (5%)** | **131 (2%)** |
| **Female** | 2,494 (40) | 1,966 (39) | 307 (47) | 165 (53) | 56 (43) |
| **Age at baseline in years, mean (SD)** | 58.0 (12.9) | 59.3 (12.6) | 50.8 (12.6) | 52.6 (11.4) | 54.0 (12.3) |
| **Age at baseline in years** |  |  |  |  |  |
| 18-49 | 1,576 (26) | 1,081 (21) | 320 (49) | 124 (39) | 51 (39) |
| 50-59 | 1,868 (30) | 1,531 (30) | 177 (27) | 120 (38) | 40 (31) |
| 60-69 | 1,530 (25) | 1,350 (27) | 113 (17) | 41 (13) | 26 (20) |
| 70+ | 1,188 (19) | 1,096 (22) | 49 (7) | 29 (9) | 14 (11) |
| **Year of second-line treatment initiation** |  |  |  |  |  |
| 2014 | 1,067 (17) | 922 (18) | 79 (12) | 46 (15) | 20 (15) |
| 2015-2016 | 2,229 (36) | 1,887 (37) | 207 (31) | 93 (30) | 42 (32) |
| 2017-2018 | 1,870 (30) | 1,480 (29) | 228 (35) | 120 (38) | 42 (32) |
| 2019-2020 | 996 (16) | 769 (15) | 145 (22) | 55 (18) | 27 (21) |
| **Months on MTF monotherapy, median (IQR)** | 21 (7-38) | 21 (7-38) | 26 (8-45) | 19 (5-40) | 14 (4-37) |
| **Years on first-line (MTF monotherapy)** |  |  |  |  |  |
| <0.5 | 1,412 (23) | 1,147 (23) | 142 (22) | 86 (27) | 37 (28) |
| 0.5-0.99 | 716 (12) | 602 (12) | 63 (10) | 31 (10) | 20 (15) |
| ≥1 | 4,034 (65) | 3,309 (65) | 454 (69) | 197 (63) | 74 (56) |
| **No. of patients registered at the person’s GP, median (IQR)** | 10306 (6643-14695) | 10562 (7051-15090) | 8537 (5182-12829) | 10267 (6400-14667) | 10306 (6312-15416) |
| **No. of patients registered at the person’s GP** |  |  |  |  |  |
| <7,000 | 1,648 (27) | 1,244 (25) | 278 (42) | 90 (29) | 36 (27) |
| 7,000-10,000 | 1,269 (21) | 1,089 (22) | 97 (15) | 58 (18) | 25 (19) |
| 10,000-15,000 | 1,749 (28) | 1,453 (29) | 170 (26) | 90 (29) | 36 (27) |
| >=15,000 | 1,496 (24) | 1,272 (25) | 114 (17) | 76 (24) | 34 (26) |
| **Hospitalisation (any) within 1 year prior to second-line treatment initiation** | 2,059 (33) | 8,818 (29) | 873 (25) | 361 (28) | 164 (30) |
| **IMD quintile** |  |  |  |  |  |
| 1 (least deprived) | 1,023 (17) | 934 (18) | 59 (9) | 9 (3) | 21 (16) |
| 2 | 1,095 (18) | 983 (19) | 80 (12) | 16 (5) | 16 (12) |
| 3 | 1,271 (21) | 1,073 (21) | 130 (20) | 49 (16) | 19 (15) |
| 4 | 1,329 (22) | 1,017 (20) | 178 (27) | 93 (30) | 41 (31) |
| 5 (most deprived) | 1,424 (23) | 1,032 (20) | 211 (32) | 147 (47) | 34 (26) |
| Missing | 20 (0) | 19 (0) | <5 (0) | <5 (0) | <5 (0) |
| **HbA1c at baseline (mmol/mol), mean (SD)** | 79 (20) | 78 (20) | 77 (19) | 85 (25) | 83 (22) |
| **HbA1c at baseline (mmol/mol)** |  |  |  |  |  |
| <53 (7%) | 130 (2) | 109 (2) | 14 (2) | 5 (2) | <5 (2) |
| 53-74 | 2,103 (34) | 1,713 (34) | 273 (41) | 85 (27) | 32 (24) |
| 75+ (9%) | 2,037 (33) | 1,657 (33) | 206 (31) | 129 (41) | 45 (34) |
| Missing | 1,892 (31) | 1,579 (31) | 166 (25) | 95 (30) | 52 (40) |
| **Blood pressure (mm Hg)** |  |  |  |  |  |
| Systolic, mean (SD) | 132 (14) | 133 (14) | 129 (14) | 131 (15) | 130 (12) |
| Diastolic, mean (SD) | 79 (9) | 79 (9) | 79 (9) | 80 (10) | 79 (8) |
| **Hypertensive, based on last recorded blood pressure** |  |  |  |  |  |
| Normotensive | 1,593 (26) | 1,242 (25) | 229 (35) | 86 (27) | 36 (27) |
| Hypertensive | 4,517 (73) | 3,771 (75) | 426 (65) | 228 (73) | 92 (70) |
| Missing | 52 (1) | 45 (1) | <5 (1) | 0 (0) | <5 (2) |
| **BMI (kg/m^2^), mean (SD)** | 32.9 (7.4) | 33.3 (7.4) | 30.1 (6.2) | 32.1 (8.8) | 30.9 (6.4) |
| **BMI category** |  |  |  |  |  |
| Under/normal weight | 267 (4) | 195 (4) | 35 (5) | 24 (8) | 13 (10) |
| Overweight | 716 (12) | 591 (12) | 81 (12) | 29 (9) | 15 (11) |
| Obese | 1,553 (25) | 1,388 (27) | 86 (13) | 52 (17) | 27 (21) |
| Missing | 3,626 (59) | 2,884 (57) | 457 (69) | 209 (67) | 76 (58) |
| **Smoking status** |  |  |  |  |  |
| Non-smoker | 672 (11) | 531 (10) | 83 (13) | 39 (12) | 19 (15) |
| Current smoker | 699 (11) | 614 (12) | 47 (7) | 26 (8) | 12 (9) |
| Ex-smoker | 1,295 (21) | 1,141 (23) | 80 (12) | 44 (14) | 30 (23) |
| Missing | 3,496 (57) | 2,772 (55) | 449 (68) | 205 (65) | 70 (53) |
| **Alcohol status** |  |  |  |  |  |
| Non-drinker | 278 (5) | 168 (3) | 63 (10) | 29 (9) | 18 (14) |
| Current drinker | 1,010 (16) | 924 (18) | 45 (7) | 27 (9) | 14 (11) |
| Ex-drinker | 506 (8) | 422 (8) | 40 (6) | 29 (9) | 15 (11) |
| Missing | 4,368 (71) | 3,544 (70) | 511 (78) | 229 (73) | 84 (64) |
| **Co-prescriptions** |  |  |  |  |  |
| RASi | 2,820 (46) | 2,418 (48) | 235 (36) | 119 (38) | 48 (37) |
| Statins | 3,859 (63) | 3,176 (63) | 426 (65) | 178 (57) | 79 (60) |
| **Cancer (any)** | 722 (12) | 659 (13) | 35 (5) | 14 (4) | 14 (11) |
| **Macrovascular comorbidities** |  |  |  |  |  |
| CVD composite* | 1,422 (23) | 1,247 (25) | 107 (16) | 47 (15) | 21 (16) |
| Amputation | 50 (1) | 47 (1) | <5 (0) | <5 (0) | <5 (1) |
| Heart failure | 365 (6) | 311 (6) | 29 (4) | 17 (5) | 8 (6) |
| Myocardial infarction | 385 (6) | 344 (7) | 30 (5) | <5 (1) | 7 (5) |
| Stroke | 343 (6) | 300 (6) | 22 (3) | 17 (5) | 4 (3) |
| Ischaemic heart disease | 1,071 (17) | 944 (19) | 87 (13) | 24 (8) | 16 (12) |
| Unstable angina | 196 (3) | 167 (3) | 21 (3) | 5 (2) | <5 (2) |
| **Microvascular comorbidities** |  |  |  |  |  |
| eGFR at baseline (mL/min/1.73m^2^), mean (SD) | 92 (19) | 91 (18) | 101 (17) | 92 (19) | 96 (18) |
| eGFR at baseline category (mL/min/1.73m^2^) |  |  |  |  |  |
| No known CKD (eGFR missing) | 171 (3) | 612 (2) | 31 (1) | 11 (1) | 21 (4) |
| 90+ (Stage 1) | 3,623 (59) | 143 (3) | 15 (2) | 5 (2) | 8 (6) |
| 60-89 (Stage 2) | 2,001 (32) | 2,856 (56) | 505 (77) | 180 (57) | 82 (63) |
| 45-59 (Stage 3a) | 275 (4) | 1,728 (34) | 125 (19) | 111 (35) | 37 (28) |
| 30-44 (Stage 3b) | 92 (1) | 248 (5) | 11 (2) | 13 (4) | <5 (2) |
| Blindness | 74 (1) | 59 (1) | 10 (2) | 5 (2) | 0 (0) |
| Hypoglycaemia | 83 (1) | 73 (1) | <5 (1) | <5 (1) | <5 (2) |
| Proteinuria | 382 (6) | 323 (6) | 34 (5) | 15 (5) | 10 (8) |
|  |  |  |  |  |  |
|  |  | | | | |

*CVD composite: heart failure, IHD, MI, stroke, unstable angina

BMI: body mass index; CPRD: Clinical Practice Research Datalink; CVD: cardiovascular; DPP4i: dipeptidyl peptidase-4 inhibitors; eGFR: estimated glomerular filtration rate; GP: general practice; HES: Hospital Episode Statistics; IMD: index of multiple deprivation; IQR: interquartile range; MTF: metformin; RASi: renin-angiotensin system inhibitors; SGTL2i: sodium-glucose co-transporter 2 inhibitors; SD: standard deviation; SU: sulfonylureas; TZD: thiazolidinediones; UK: United Kingdom

**Supplementary table 4:** Summary of missing covariate data in people excluded from the main complete case analysis.

| **Covariate with missing data** | **Overall**  **42,533** | **White**  **35,801** | **South Asian**  **4,117** | **Black**  **1,588** | **Mixed/other**  **679** |
| --- | --- | --- | --- | --- | --- |
| **Ethnicity** | 348 (1) | - | - | - | - |
| **IMD quintile** | 20 (0) | 19 (0) | <5 (0) | 0 (0) | 0 (0) |
| **HbA1c** | 1,892 (4) | 1,579 (4) | 166 (4) | 95 (6) | 52 (8) |
| **Blood pressure status** | 52 (0) | 45 (0) | <5 (0) | 0 (0) | <5 (0) |
| **BMI** | 3,626 (9) | 2,884 (8) | 457 (11) | 209 (13) | 76 (11) |
| **Smoking status** | 3,496 (8) | 2,772 (8) | 449 (11) | 205 (13) | 70 (10) |
| **Alcohol status** | 4,368 (10) | 3,544 (10) | 511 (12) | 229 (14) | 84 (12) |

**Supplementary table 5:** Adjusted predicted percentages of second-line treatment prescribed by ethnicity and IMD.

| **Second-line antidiabetic prescribed** | **Ethnicity or IMD quintile** | **^1^Adjusted predicted probability (95% CI)** | **P-value (Wald test)** | **P-value (joint-test)** |
| --- | --- | --- | --- | --- |
| Ethnicity |  |  |  |  |
| SU | White | 0.36 (0.34-0.37) | - |  |
|  | South Asian | 0.37 (0.34-0.39) | 0.37 |  |
|  | Black | 0.37 (0.34-0.40) | 0.25 |  |
|  | Mixed/other | 0.36 (0.32-0.40) | 0.96 | 0.61 |
| DPP4i | White | 0.43 (0.40-0.45) | - |  |
|  | South Asian | 0.44 (0.41-0.47) | 0.47 |  |
|  | Black | 0.44 (0.40-0.48) | 0.40 |  |
|  | Mixed/other | 0.47 (0.42-0.51) | 0.05 | 0.21 |
| SGLT2i | White | 0.21 (0.19-0.23) | - |  |
|  | South Asian | 0.20 (0.18-0.22) | 0.04 |  |
|  | Black | 0.19 (0.16-0.22) | 0.02 |  |
|  | Mixed/other | 0.17 (0.14-0.21) | 0.01 | 0.003 |
| IMD quintile |  |  |  |  |
| SU | 1 (least deprived) | 0.36 (0.34-0.38) | - |  |
|  | 2 | 0.35 (0.33-0.37) | 0.15 |  |
|  | 3 | 0.36 (0.34-0.38) | 0.78 |  |
|  | 4 | 0.36 (0.34-0.38) | 0.87 |  |
|  | 5 (most deprived) | 0.36 (0.35-0.38) | 0.53 | 0.26 |
| DPP4i | 1 (least deprived) | 0.42 (0.39-0.45) | - |  |
|  | 2 | 0.43 (0.40-0.46) | 0.07 |  |
|  | 3 | 0.42 (0.40-0.45) | 0.33 |  |
|  | 4 | 0.43 (0.40-0.46) | 0.14 |  |
|  | 5 (most deprived) | 0.44 (0.42-0.47) | 0.003 | 0.04 |
| SGLT2i | 1 (least deprived) | 0.22 (0.20-0.25) | - |  |
|  | 2 | 0.22 (0.20-0.24) | 0.65 |  |
|  | 3 | 0.22 (0.20-0.24) | 0.40 |  |
|  | 4 | 0.21 (0.19-0.23) | 0.05 |  |
|  | 5 (most deprived) | 0.19 (0.17-0.21) | <0.001 | <0.001 |

**^1^**Mutually adjusted for deprivation (ethnicity estimates) and ethnicity (deprivation estimates), as well as number of patients registered at the patients’ GP practice, years on first-line category, age category, sex, last HbA1c prior to second-line initiation category, BMI, prevalent heart failure, ischaemic heart disease, myocardial infarction, stroke, unstable angina, RASi and/or statin co-prescription, CKD category, blood pressure category, history of proteinuria, blindness, cancer (any), hospitalisation (any) in past year, smoking status, alcohol status, region, all as fixed effects, and CCG-clustering as a random effect.

**Supplementary table 6:** Results from the multinomial, multivariable logistic regression model used to calculate adjusted predicted percentages for the association between ethnicity, deprivation, and second-line antidiabetic treatment prescribed

| **Second-line antidiabetic prescribed** | **Ethnicity or IMD quintile** | **Age and sex adjusted,**  **OR (95% CI)** | **^1^Fixed effect model,**  **OR (95% CI)** | **^2^Random effect model,**  **OR (95% CI)** |
| --- | --- | --- | --- | --- |
| **Ethnicity** | | | | |
| DPP4i vs. SU (base) | White | 1 | 1 | 1 |
|  | South Asian | 1.07 (0.99-1.16) | 0.97 (0.88-1.06) | 0.99 (0.89-1.10) |
|  | Black | 0.79 (0.70-0.90) | 0.88 (0.77-1.01) | 0.98 (0.84-1.13) |
|  | Mixed/other | 1.04 (0.86-1.25) | 1.04 (0.85-1.27) | 1.10 (0.89-1.35) |
| SGLT2i vs. SU (base) | White | 1 | 1 | 1 |
|  | South Asian | 0.76 (0.69-0.84) | 0.93 (0.82-1.05) | 0.88 (0.77-1.01) |
|  | Black | 0.46 (0.39-0.55) | 0.69 (0.57-0.84) | 0.79 (0.64-0.97) |
|  | Mixed/other | 0.68 (0.53-0.87) | 0.77 (0.59-1.02) | 0.76 (0.57-1.01) |
| SGLT2i vs. DPP4i (base) | White | 1 | 1 | 1 |
|  | South Asian | 0.71 (0.64-0.78) | 0.96 (0.86-1.08) | 0.90 (0.79-1.01) |
|  | Black | 0.58 (0.49-0.70) | 0.79 (0.65-0.95) | 0.81 (0.67-0.99) |
|  | Mixed/other | 0.65 (0.51-0.83) | 0.75 (0.58-0.96) | 0.70 (0.54-0.91) |
| **IMD quintile** |  |  |  |  |
| DPP4i vs. SU (base) | 1 (least deprived) | 1 | 1 | 1 |
|  | 2 | 1.02 (0.94-1.10) | 1.03 (0.95-1.13) | 1.08 (0.99-1.19) |
|  | 3 | 0.99 (0.91-1.07) | 0.97 (0.89-1.06) | 1.03 (0.94-1.13) |
|  | 4 | 0.99 (0.92-1.07) | 1.04 (0.95-1.13) | 1.03 (0.94-1.13) |
|  | 5 (most deprived) | 1.08 (1.00-1.16) | 1.10 (1.01-1.20) | 1.05 (0.95-1.16) |
| SGLT2i vs. SU (base) | 1 (least deprived) | 1 | 1 | 1 |
|  | 2 | 0.95 (0.86-1.05) | 0.96 (0.86-1.07) | 1.02 (0.91-1.15) |
|  | 3 | 0.89 (0.81-0.98) | 0.92 (0.83-1.03) | 0.97 (0.87-1.09) |
|  | 4 | 0.80 (0.73-0.87) | 0.89 (0.80-0.99) | 0.91 (0.81-1.03) |
|  | 5 (most deprived) | 0.71 (0.65-0.78) | 0.80 (0.71-0.89) | 0.80 (0.71-0.91) |
| SGLT2i vs. DPP4i (base) | 1 (least deprived) | 1 | 1 | 1 |
|  | 2 | 0.98 (0.91-1.06) | 0.93 (0.84-1.03) | 0.94 (0.85-1.05) |
|  | 3 | 1.01 (0.94-1.09) | 0.95 (0.86-1.05) | 0.94 (0.85-1.05) |
|  | 4 | 1.01 (0.94-1.09) | 0.86 (0.78-0.95) | 0.88 (0.79-0.98) |
|  | 5 (most deprived) | 0.93 (0.86-1.00) | 0.72 (0.65-0.80) | 0.75 (0.67-0.85) |
| ^1^Adjusted for IMD quintile (for ethnicity estimates), ethnicity (for IMD quintile estimates), no. of patients registered at the person’s GP, years on first-line category, age category, sex, last HbA1c prior to second-line initiation category, BMI, prevalent heart failure, ischaemic heart disease, myocardial infarction, stroke, unstable angina, RASi and/or statin co-prescription, CKD category, blood pressure category, history of proteinuria, blindness, cancer (any), hospitalisation (any) in past year, smoking status, alcohol status, region.  ^2^Adjusted for all covariates above, as well as CCG clustering using random effects. | | | | |

**Supplementary table 7:** Adjusted predicted percentages of second-line treatment choice by ethnicity and IMD, stratified by prevalent CVD status.

| **Outcome** | **Ethnicity or IMD quintile** | **No prevalent CVD,**  **Adjusted predicted percentage (95% CI)** | **Prevalent CVD,**  **Adjusted predicted percentage (95% CI)** | **P-value (Wald test)** |
| --- | --- | --- | --- | --- |
| **Ethnicity** |  |  |  |  |
| SU | White | 0.36 (0.34-0.37) | 0.36 (0.34-0.38) | 0.13 |
|  | South Asian | 0.36 (0.34-0.39) | 0.37 (0.35-0.40) | 0.20 |
|  | Black | 0.37 (0.34-0.40) | 0.38 (0.35-0.41) | 0.28 |
|  | Mixed/other | 0.36 (0.32-0.40) | 0.36 (0.32-0.40) | 0.47 |
| DPP4i | White | 0.42 (0.39-0.44) | 0.46 (0.44-0.49) | <0.001 |
|  | South Asian | 0.42 (0.39-0.46) | 0.47 (0.44-0.50) | <0.001 |
|  | Black | 0.43 (0.39-0.47) | 0.47 (0.44-0.51) | <0.001 |
|  | Mixed/other | 0.46 (0.41-0.50) | 0.50 (0.45-0.55) | <0.001 |
| SGLT2i | White | 0.23 (0.21-0.25) | 0.17 (0.15-0.19) | <0.001 |
|  | South Asian | 0.21 (0.19-0.24) | 0.16 (0.14-0.18) | <0.001 |
|  | Black | 0.20 (0.17-0.23) | 0.15 (0.12-0.17) | <0.001 |
|  | Mixed/other | 0.19 (0.15-0.22) | 0.14 (0.11-0.17) | <0.001 |
| **IMD quintile** |  |  |  |  |
| SU | 1 (least deprived) | 0.36 (0.34-0.38) | 0.37 (0.34-0.39) | 0.11 |
|  | 2 | 0.35 (0.33-0.37) | 0.35 (0.33-0.38) | 0.15 |
|  | 3 | 0.36 (0.34-0.37) | 0.36 (0.34-0.39) | 0.14 |
|  | 4 | 0.36 (0.34-0.38) | 0.37 (0.35-0.39) | 0.18 |
|  | 5 (most deprived) | 0.36 (0.34-0.38) | 0.37 (0.35-0.39) | 0.30 |
| DPP4i | 1 (least deprived) | 0.41 (0.38-0.43) | 0.45 (0.42-0.48) | <0.001 |
|  | 2 | 0.42 (0.39-0.45) | 0.47 (0.44-0.50) | <0.001 |
|  | 3 | 0.41 (0.39-0.44) | 0.46 (0.43-0.49) | <0.001 |
|  | 4 | 0.42 (0.39-0.45) | 0.46 (0.44-0.49) | <0.001 |
|  | 5 (most deprived) | 0.43 (0.41-0.46) | 0.48 (0.45-0.50) | <0.001 |
| SGLT2i | 1 (least deprived) | 0.24 (0.21-0.26) | 0.18 (0.16-0.20) | <0.001 |
|  | 2 | 0.23 (0.21-0.26) | 0.18 (0.16-0.20) | <0.001 |
|  | 3 | 0.23 (0.21-0.25) | 0.18 (0.16-0.20) | <0.001 |
|  | 4 | 0.22 (0.20-0.24) | 0.17 (0.15-0.19) | <0.001 |
|  | 5 (most deprived) | 0.20 (0.18-0.23) | 0.15 (0.14-0.17) | <0.001 |

**Supplementary table 8:** Describing the potential mediating effect of deprivation on the association between ethnicity and second-line antidiabetic treatment prescribed using results from multivariable, multinomial logistic regression models

| **Second-line antidiabetic prescribed** | **Ethnicity** | **^1^Partially adjusted random effect model,**  **OR (95% CI)** | **^2^Adjusted random effect model,**  **OR (95% CI)** |
| --- | --- | --- | --- |
| DPP4i vs. SU (base) | White | 1 | 1 |
|  | South Asian | 0.97 (0.88-1.06) | 0.99 (0.89-1.10) |
|  | Black | 0.88 (0.77-1.01) | 0.98 (0.84-1.13) |
|  | Mixed/other | 1.04 (0.85-1.27) | 1.10 (0.89-1.35) |
| SGLT2i vs. SU (base) | White | 1 | 1 |
|  | South Asian | 0.93 (0.82-1.05) | 0.88 (0.77-1.01) |
|  | Black | 0.69 (0.57-0.84) | 0.79 (0.64-0.97) |
|  | Mixed/other | 0.77 (0.59-1.02) | 0.76 (0.57-1.01) |
| SGLT2i vs. DPP4i (base) | White | 1 | 1 |
|  | South Asian | 0.96 (0.86-1.08) | 0.90 (0.79-1.01) |
|  | Black | 0.79 (0.65-0.95) | 0.81 (0.67-0.99) |
|  | Mixed/other | 0.75 (0.58-0.96) | 0.70 (0.54-0.91) |

^1^Adjusted for no. of patients registered at the person’s GP, years on first-line category, age category, sex, last HbA1c prior to second-line initiation category, BMI, prevalent heart failure, ischaemic heart disease, myocardial infarction, stroke, unstable angina, RASi and/or statin co-prescription, CKD category, blood pressure category, history of proteinuria, blindness, cancer (any), hospitalisation (any) in past year, smoking status, alcohol status, region, and CCG clustering (as a random effect).

^2^Additionally adjusted for IMD quintile, as well as all covariates and CCG clustering as above.

**Supplementary table 9:** Second-line antidiabetic treatment prescribed stratified by time, crude proportions, n (row %)

|  | **Second-line antidiabetic treatment prescribed** | | |
| --- | --- | --- | --- |
| **Year of second-line treatment initiation** | **SU** | **DPP4i** | **SGLT2i** |
| 2014 | 2,682 (63) | 1,323 (31) | 244 (6) |
| 2015-16 | 5,138 (46) | 4,596 (41) | 1,433 (13) |
| 2017-18 | 3,728 (30) | 5,968 (48) | 2,739 (22) |
| 2019-20 | 2,161 (24) | 3,814 (43) | 2,978 (33) |

**Supplementary table 10:** Adjusted predicted percentages of second-line treatment prescribed by year of second-line treatment initiation, overall and stratified by prevalent CVD status.

| **Outcome** | **Year of second-line initiation** | **Overall, adjusted predicted probability (95% CI)** | **No prevalent CVD,**  **Adjusted predicted probability (95% CI)** | **Prevalent CVD,**  **Adjusted predicted probability (95% CI)** |
| --- | --- | --- | --- | --- |
| SU | 2014 | 0.60 (0.57-0.62) | 0.60 (0.58-0.62) | 0.60 (0.57-0.63) |
|  | 2015-16 | 0.45 (0.43-0.47) | 0.45 (0.42-0.47) | 0.45 (0.43-0.48) |
|  | 2017-18 | 0.29 (0.27-0.31) | 0.29 (0.27-0.31) | 0.30 (0.28-0.32) |
|  | 2019-20 | 0.23 (0.21-0.24) | 0.22 (0.21-0.24) | 0.24 (0.22-0.26) |
| DPP4i | 2014 | 0.34 (0.31-0.36) | 0.33 (0.30-0.36) | 0.35 (0.32-0.38) |
|  | 2015-16 | 0.41 (0.39-0.44) | 0.41 (0.38-0.43) | 0.44 (0.41-0.47) |
|  | 2017-18 | 0.48 (0.45-0.51) | 0.47 (0.44-0.49) | 0.52 (0.49-0.54) |
|  | 2019-20 | 0.43 (0.40-0.46) | 0.41 (0.38-0.44) | 0.48 (0.45-0.51) |
| SGLT2i | 2014 | 0.07 (0.05-0.08) | 0.07 (0.06-0.08) | 0.05 (0.04-0.06) |
|  | 2015-16 | 0.14 (0.12-0.16) | 0.45 (0.13-0.17) | 0.11 (0.09-0.12) |
|  | 2017-18 | 0.23 (0.21-0.25) | 0.24 (0.22-0.27) | 0.18 (0.16-0.21) |
|  | 2019-20 | 0.34 (0.32-0.37) | 0.36 (0.33-0.39) | 0.28 (0.26-0.31) |

**Supplementary table 11:** Results from the multinomial, multivariable logistic regression model used to calculate adjusted predicted percentages for the association between year of second-line treatment initiation and second-line antidiabetic treatment prescribed

| **Second-line antidiabetic prescribed** | **Predictor variable** | **Age and sex adjusted,**  **OR (95% CI)** | **^1^Fixed effect model,**  **OR (95% CI)** | **^2^Random effect model,**  **OR (95% CI)** |
| --- | --- | --- | --- | --- |
| **Year of second-line initiation** | | | | |
| DPP4i vs. SU (base) | 2014 | 1 | 1 | 1 |
|  | 2015-16 | 1.79 (1.66-1.94) | 1.81 (1.67-1.96) | 1.77 (1.63-1.93) |
|  | 2017-18 | 3.21 (2.97-3.47) | 3.31 (3.04-3.59) | 3.32 (3.04-3.62) |
|  | 2019-20 | 3.51 (3.22-3.82) | 3.77 (3.44-4.12) | 3.79 (3.44-4.17) |
| SGLT2i vs. SU (base) | 2014 | 1 | 1 | 1 |
|  | 2015-16 | 3.16 (2.73-3.66) | 3.33 (2.87-3.88) | 3.38 (2.89-3.95) |
|  | 2017-18 | 8.52 (7.38-9.84) | 9.75 (8.40-11.3) | 10.1 (8.65-11.78) |
|  | 2019-20 | 16.4 (14.2-19.0) | 20.4 (17.5-23.8) | 22.5 (19.2-26.4) |
| SGLT2i vs. DPP4i (base) | 2014 | 1 | 1 | 1 |
|  | 2015-16 | 1.76 (1.51-2.05) | 1.84 (1.56-2.15) | 1.91 (1.63-2.24) |
|  | 2017-18 | 2.65 (2.29-3.08) | 2.95 (2.54-3.43) | 3.05 (2.61-3.56) |
|  | 2019-20 | 4.69 (4.04-5.44) | 5.42 (4.65-6.31) | 5.95 (5.08-6.96) |
| ^1^Adjusted for no. of patients registered at the person’s GP, years on first-line category, age category, sex, last HbA1c prior to second-line initiation category, BMI, prevalent heart failure, ischaemic heart disease, myocardial infarction, stroke, unstable angina, RASi and/or statin co-prescription, CKD category, blood pressure category, history of proteinuria, blindness, cancer (any), hospitalisation (any) in past year, smoking status, alcohol status, region.  ^2^Adjusted for all covariates above, as well as CCG clustering using random effects. | | | | |

**Supplementary figure 1:** Proportion of people prescribed each second-line antidiabetic between 2014-2020, overall and stratified by prevalent CVD at baseline status.


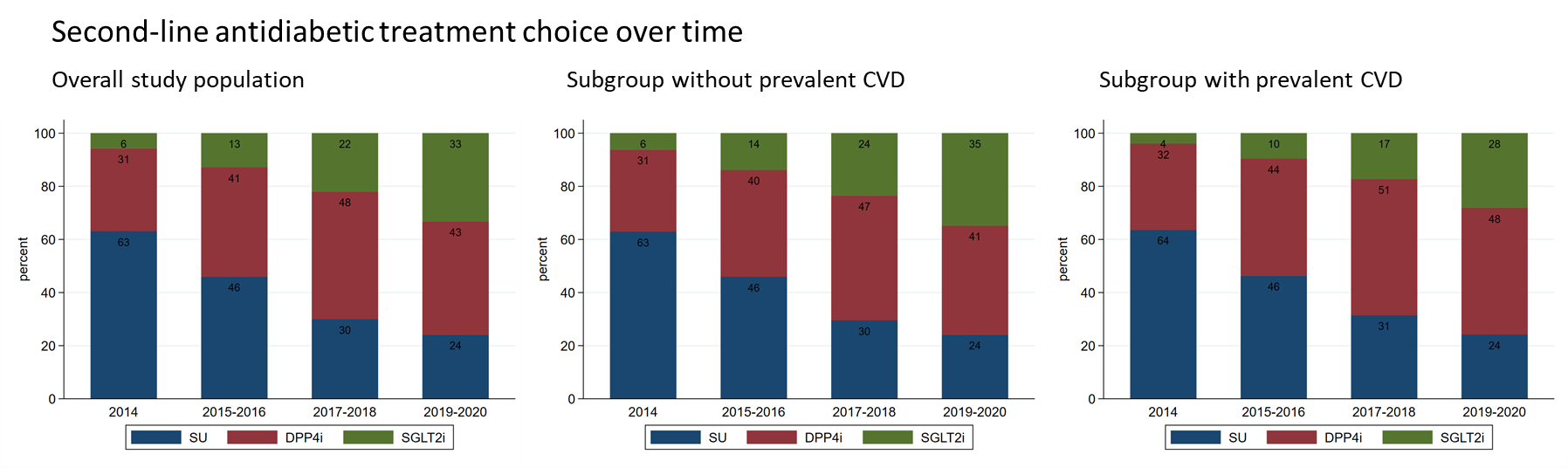

Supplement: Supplementary file 1 — Appendix S1: Supporting information [file DOM-25-282-s001.docx]
